# Hand hygiene during the spread of COVID-19: a cross-sectional study of awareness and practices among academic institutions in Lebanon

**DOI:** 10.3389/fpubh.2023.1256433

**Published:** 2024-01-05

**Authors:** Nisreen Alwan, Jihan Safwan, Racha Kerek, Wissam Ghach

**Affiliations:** ^1^College of Health Sciences, Abu Dhabi University, Abu Dhabi, United Arab Emirates; ^2^School of Pharmacy, Lebanese International University, Beirut, Lebanon; ^3^Institut National de Santé Publique, d’Épidémiologie Clinique et de Toxicologie-Liban (INSPECT-LB), Beirut, Lebanon; ^4^Faculty of Health Sciences, Lebanese University, Tripoli, Lebanon; ^5^Department of Natural Sciences, Lebanese American University, Byblos, Lebanon; ^6^Department of Public Health, Canadian University Dubai, Dubai, United Arab Emirates; ^7^School of Health Sciences, Modern University for Business and Science, Beirut, Lebanon

**Keywords:** COVID-19, awareness, practices, hand hygiene, university community, Lebanon

## Abstract

**Introduction:**

During the pandemic, the World Health Organization has recommended hand hygiene as one of the effective preventive measures to limit the global spread of COVID-19. However, the awareness gap of hand hygiene protocols could increase the spread of COVID-19 and consequently increase the absenteeism rate among academic institutions. This study aims to assess hand hygiene awareness and practices levels among various university communities in Lebanon.

**Methods:**

A cross-sectional study was conducted between December 2021 and June 2022 among 1,291 participants from academic settings in Lebanese universities. An online survey (score-based questionnaire) of hand hygiene was conducted to evaluate the awareness and practices among university communities (faculty, staff, and students). Mann-Whitney and Kruskal-Wallis tests were used to determine whether significant differences exist in the levels of awareness with regard to gender, age, provinces, educational level, and university status. Pearson’s chi-squared test was applied to assess differences among the sample characteristics and participants’ practice of hand hygiene.

**Results:**

It was found that most of the participants showed a moderate level of awareness (76.4%) with a mean score of 7.59 out of 12 (SD = 1.68). The Mann-Whitney test indicated that females recorded higher levels of awareness than males with a significant difference of 102, 104: *p* < 0.05. Another notable variable was the educational level of the participants with university degrees holders recording higher scores of awareness than the ones with high school degrees as per the Kruskal-Wallis test (*p* < 0.05). Significant differences were also shown in awareness scores among the age groups and the university status (*p* < 0.05). The Pearson’s chi-squared test results showed that females used alcohol-based hand rubs or soap and water more frequently than males (*p* < 0.05). However, males significantly preferred the frequent use of water alone compared to females (*p* < 0.05).

**Conclusion:**

The study findings highlighted the necessity of awareness campaigns and health educational programs addressing the technical skills of hand hygiene among both genders (especially males) of the academic communities in Lebanon.

## 1 Introduction

The Coronavirus Disease 2019 (COVID-19) pandemic, caused by the novel coronavirus SARS-CoV-2, has caused over 765 million cases and 6 million deaths globally (1). The COVID-19 infection is characterized by having a wide spectrum of symptoms ranging from respiratory to extrapulmonary ones and may lead to lung failure and death (2, 3). The virus can spread by human-to-human interactions via coughing, sneezing, respiratory droplets, and aerosols from an infected person especially in poorly ventilated and/or crowded indoor settings and human-to-object interactions by touching their eyes, nose, or mouth upon contacting surfaces that have been contaminated by the virus (4). To prevent its spread, the World Health Organization (WHO) has taken immediate measures and drastic precautions since the onset of the pandemic. Hand disinfection by soap washing or disinfectants indeed is one of the most effective ways to reduce the spread of the novel coronavirus through deactivating the virus which is translocated from contaminated surfaces (5).

According to studies conducted prior and during the COVID-19 pandemic, hand hygiene was and is still considered essential for reducing the spread of infectious diseases in various settings including universities (6–16). Despite that, it was found that university students improperly wash their hands thus increasing their chances of contracting and spreading infectious diseases and having high absenteeism (6, 12, 17). In 2011, a study was conducted to evaluate the Turkish University students’ social handwashing knowledge, practices, skills, and related factors. The results showed that around 27% of the participants wash their hands less than five times a day. The main reason for skipping handwashing was the participants’ belief of the ‘no need’ to wash their hands (6). A cross-sectional study carried out on undergraduate medical, dental, and nursing students in a Tertiary Care Teaching Institute in Navi Mumbai, India has indicated that 69.1% of the participants had moderate knowledge concerning hand hygiene stressing on the need to focus more on hand hygiene education among undergraduate healthcare students and improve primary training as well as curricula (7). Sultana et al. conducted a study involving university students in Dhaka, Bangladesh. The results revealed the low awareness levels and inadequate hand hygiene practices among students (18). Sallami assessed hand hygiene knowledge, attitude, and practice among health science students in Aden University in Yemen. The results revealed good levels in the three aspects but with an obvious lack of knowledge on the main source and route of cross-contamination with pathogens in hospital settings (9).

A study on the knowledge and practices of hand hygiene between 1st and 3rd year nursing students from selected institutions in Ganktok, Sikkim showed that they had average knowledge and good practice with correlation between the two. There was also significant association between practices of hand hygiene and age, current year, and parents’ occupational status (15). At a larger Midwestern University, students were noticed washing their hands inadequately, which would increase their risks of contracting infectious diseases (19). In 2019, Mbouthieu Teumta et al. conducted a study to evaluate handwashing knowledge, practices, and skills of students in both private and public institutions of higher learning in Bamenda, Cameroon (20). Around 75% of the study participants had poor handwashing practices scores and 56.6% of the participants washed their hands less than six times a day. This shows that there are gaps in hand hygiene knowledge and practices among university settings that must be addressed especially in the period of the COVID pandemic where infections could increase the students’ absenteeism and eventually affect their learning abilities (20). Olorunpomi et al. have evaluated students’ understanding and practice of hand hygiene at Adeleke University in Nigeria which showed that most respondents had good knowledge and practice of hand hygiene. Despite that, absence of soap and detergents was a major hurdle in this study (14).

Based on what has been stated, a limited number of studies were conducted targeting this aspect and addressing only university students with two in the Middle East (6, 9). Surprisingly, none of these were carried out during the COVID-19 period. In Lebanon, a single study measured the levels of awareness and performance toward COVID-related disinfectant use among the university communities in Lebanon to find out that these communities had a weak level of awareness, a moderate level of performance, and a weak correlation between the awareness and performance levels (21). Another study was conducted on the public community of Lebanon to find out that the public community had a lower level of awareness than performance regarding the safe use of disinfectants and household cleaners during the spread of COVID-19 in Lebanon (22). These studies were limited on investigating the community awareness regarding the use of chemical-based disinfectants and cleaning products as well as to what extent the community practices (including hand washing) are appropriate and safe rather than evaluating their awareness and practices toward the safe protocols of hand hygiene for COVID-19 prevention. Additionally, these literature studies highlighted the poor awareness regarding the hygiene-based preventive measures among several communities in Lebanon during the COVID-19 period, and highlighted that there is a literature gap in hand hygiene awareness and practices among university communities specifically in Lebanon as one of the Middle Eastern countries that must be addressed especially during the spread of COVID pandemic where infections could increase students’ absenteeism and eventually affect their learning abilities. The aim of this study is to assess hand hygiene awareness and practices among various university communities in Lebanon during the spread of COVID-19. Furthermore, this research will provide an insight for public health communities to design their future initiatives and spread the awareness of hand hygiene in case of the occurrence of alike pandemic.

## 2 Materials and methods

Using a convenient sampling strategy, this cross-sectional study was conducted between December 2021 and June 2022 to evaluate the levels of awareness and practices regarding hand hygiene among the university communities (students, staff, and faculty) during the prevention of COVID-19 in Lebanon. The validated questionnaire, prepared by Mahdi et al. (23), was posted on social media platforms (Facebook LinkedIn, Instagram, and Twitter) using google survey and shared through emails with the research departments and center of the academic institutions in Lebanon.

### 2.1 Population

A total of 1,291 individuals aged ≥18 years old from five Lebanese universities (Modern University for Business and Science, Lebanese University, Lebanese International University, Jinan University, and Lebanese American University) participated electronically in this study. The sample population was stratified into gender, age, university department, study program, and the participant’s status at the academic institution (staff, faculty, or student). The exclusion criteria included non-adult participants (aged less than 18 years old) and who were not registered at one of the Lebanese universities during the spread of COVID-19 in Lebanon.

### 2.2 Study tool

The structure of the study tool included: (1) a profile section with eight items of age, gender, educational level, province, and university status (student, staff, or faculty); (2) sources of information about the preventive measures of COVID-19; (3) 12 Awareness-related items (Yes/No) specifically discussing the risks, practices, and common misconceptions associated with hand hygiene; (4) 12 Practice-related items regarding the safe use of hand hygiene products (water only, soap and water, and alcohol-based hand rubs (ABHRs)) during the spread of COVID-19 in Lebanon.

To measure the level of awareness, each survey item answered correctly was assigned 1 point (0 point for incorrect responses), then the sum of awareness scores was calculated individually using a scale from 0 to 12, with a higher score indicating considerable awareness level on hand hygiene and a lower score indicating poor awareness. Then, the median score of awareness was then divided into intervals: weak [0, 5], moderate [6, 9], and good [10–12].

### 2.3 Study analysis

The Statistical Package for the Social Sciences, version 21 (SPSS, International Business Machine Corp. IBM, Chicago, IL, USA) was utilized to analyze the study data. To reflect the sociodemographic profile of the participants, percentage frequency was considered. The mean (or median with Interquartile range, IQR) ± standard deviation (SD) was used to summarize continuous variables including total hand hygiene awareness score. The levels and scores of awareness were represented by descriptive analyses in the study tables. Mann-Whitney test was used to determine if significant mean differences exist with regard to gender. Kruskal-Wallis test was used to check for mean differences with regard to all other variables. Prior to that, normality and homogeneity of variances were tested using Kolmogorov-Smirnov and Levene’s tests, respectively, for all variables rendering violations in these two assumptions with *p* < 0.05. Pearson’s chi-squared test was applied to assess differences among the sample characteristics and participants’ practice of hand hygiene. All data analysis was carried out at a significance level of 0.05.

### 2.4 Ethical considerations

The Institutional Review Board (IRB) at the Modern University for Business and Science (MUBS) approved the ethical application (MU20210924-25) of the study protocol. The google survey included an informed consent to identify the purpose, risks, benefits, and confidentiality of the study as well as the informatory statement “Participation is voluntary, and the submission of the questionnaire indicates your consent to participate in the study.”

## 3 Results

Out of 1,291 participants, 84.4% of the study population were young adults (aged 18–29 years old) with a mean age of 23.8 (SD = 7.32) and 63.5% were female with a sex ratio (M:F) of 1:1.75. Most of the study population (83.5%) were residents of the five major Lebanese provinces (Beirut, Mount Lebanon, Beqaa, South Lebanon, and North Lebanon). In terms of the educational level, almost half of the study population were students (89.1%) at the undergraduate level (57.9%) within several fields of study as represented in Table 1.

**Table 1:** Frequencies and percentages of participants’ characteristics (gender, age, province, educational level, and university status) at the Lebanese universities.

Variables	Frequency	%
Gender		
Male	470	36.4%
Female	821	63.6%
Age-Median (range, minimum, maximum) 21 (56.00, min.: 18; max: 74)		
18–29	1,090	84.4%
30–39	144	11.2%
40–49	36	2.8%
50–64	17	1.3%
65–74	4	0.3%
Province		
Beirut	292	22.6%
Mount Lebanon	242	18.7%
Beqaa	151	11.7%
North Lebanon	166	12.8%
South Lebanon	228	17.7%
Baalbek – Hermel	40	3.1%
Akkar	71	5.5%
Nabatieh	101	7.8%
Educational level		
High school	274	21.2%
Bachelor degree	748	57.9%
Master’s degree	226	17.5%
Pharm.D and Ph.D. degrees/Postdoctoral fellowship	43	3.3%
University status		
Student	1,150	89.1%
Staff	43	3.3%
Faculty	98	7.6%

### 3.1 Sources of COVID-19 information

**Figure 1:**
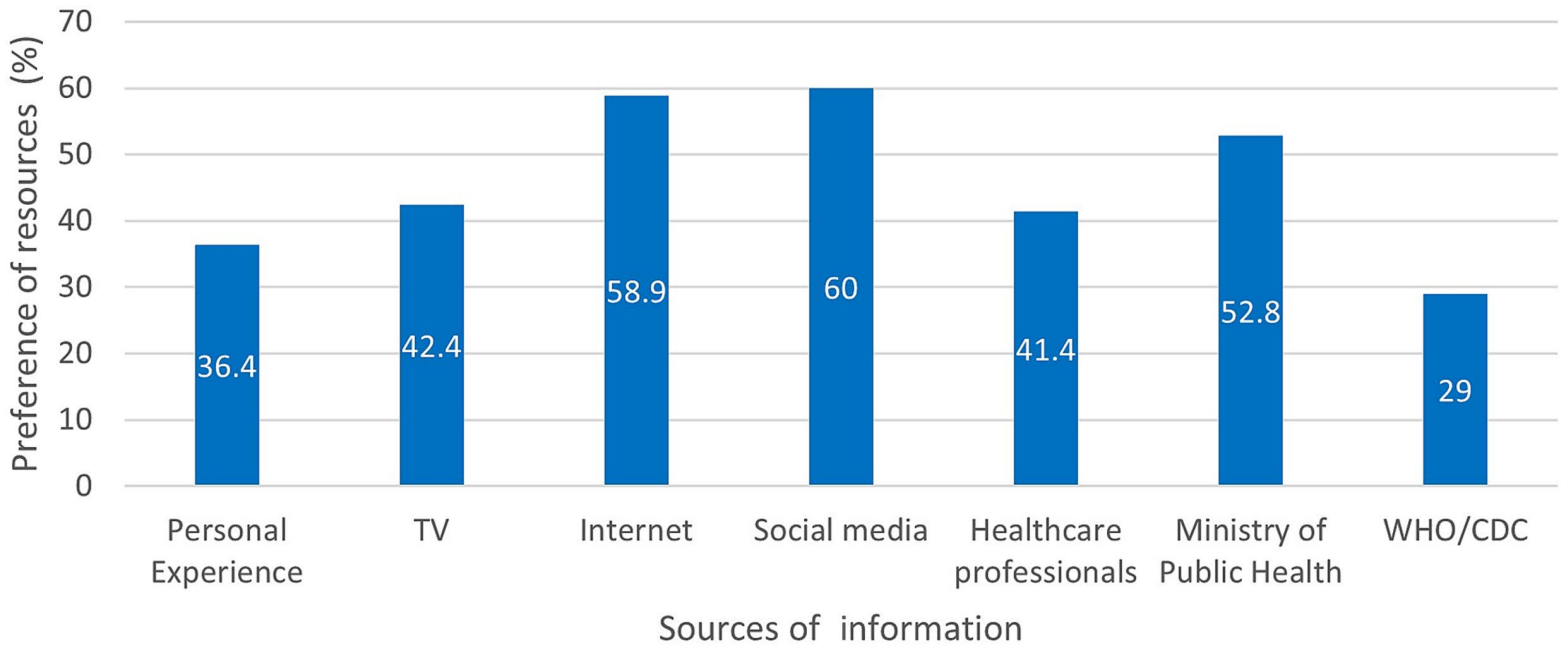
Bar graph distribution of the participants’ reliance on the sources of information regarding COVID-19 in terms of percent frequency.

Looking at the sources of COVID-19 regarding the preventive measures of COVID-19 among the study population (Figure 1), participants showed highest reliance on social media platforms (60%) and internet search engines (58.9%) while lowest reliance on the international organizations such as WHO and CDC (29%).

### 3.2 Evaluation of awareness (A)

The results of the participants’ awareness of hand hygiene are represented in Table 2. The mean score of hand hygiene awareness was 7.59 (SD = 1.68) where the majority of the study population (76.4%) scored within the moderate level of awareness (6 points < sum of awareness scores <9 points). The study population showed the lowest awareness of handwashing protocols (A6-A7 with a score of 30.9 and 47.7%, respectively) and the effectiveness of antiseptic soap (A8: 29.1%) while the highest awareness of probability of COVID-19 transmission via hands (A1 and A10 with a score of 86.3 and 89.5%, respectively). To a lesser extent, the study population was aware about the absence of the impact of hand hygiene on AIDS/HIV transmission (A2), the impact of hand hygiene on body defense mechanism (A3), the ineffectiveness of the ABHR with less than 60% alcohol for hand disinfection (A4), the ineffectiveness of the ABHR when hands are dirty (A9), and the inappropriateness of UV lamps and surface disinfectants for skin disinfection (A11-12).

**Table 2:** Frequencies and percent frequencies of the participants’ awareness regarding hand hygiene.

Awareness^*^		Correct responses	
		Frequency	Percentage
A1 – Respiratory infections including “COVID-19” can be transmitted by poor hand hygiene (**Yes**, No).		1,114	86.3%
A2 – HIV/AIDS can be transmitted by poor hand hygiene (Yes,**No**).		965	74.7%
A3 – Always keeping your hands clean may lower your body defense mechanism (Yes,**No**).		749	58%
A4- ABHR containing less than 60% of alcohol is sufficient for hands disinfection (Yes,**No**).		786	60.9%
A5 – Washing off with soap and water from 20 to 40 s is sufficient for hands disinfection (**Yes**, No).		1,064	82.4%
A6 – Hands should be held underwater while lathering with soap (**Yes**, No).		399	30.9%
A7 – The temperature of the water does not make difference in terms of the cleansing effect of handwashing (**Yes**, No).		616	47.7%
A8 – Antiseptic/antibacterial soaps are more effective at preventing illness than washing with plain soap and water (Yes,**No**).		376	29.1%
A9 – Using ABHR may not be as effective as the use of soap and water when hands are visibly dirty (**Yes**, No).		961	74.4%
A10 – There is no need to clean our hands after sneezing, coughing, or shaking hands (Yes,**No**).		1,156	89.5%
A11 – Ultra-violet (UV) lamps should not be used to disinfect our hands or other areas of our skin (**Yes**, No).		858	66.5%
A12 – Spraying and introducing bleach or another surface disinfectant into our hands is safe and it will protect us against COVID-19 and other infections (Yes,**No**).		753	58.3%
Awareness levels	Poor (score < 6 points)	137	10.6%
	Medium (6 < score < 9 points)	986	76.4%
	Good (score ≥ 10 points)	168	13%

^*^Values in bold indicate correct answers.

In Table 3, the statistical analysis of the participants’ awareness of hand hygiene showed that females had significantly (*p* < 0.05) a higher median score than males (however, age groups had a significant difference) (*p* < 0.05) without a clear relationship with the variation of age. Another notable variable was the education of the study population where the participants with education level (Bachelor, Master, Pharm.D, Ph.D., and postdoctoral fellowships) recorded significantly (*p* < 0.05) a higher median score than the high school level. In this study, the university status aligns in the same direction with the education level where faculty (participants who are expected to have the highest level of education) showed significantly (*p* < 0.05) a higher median score than students and staff. On the other hand, there was no significant difference (*p* > 0.05) among the participants when the variable of residence (Lebanese provinces) was investigated.

**Table 3:** Statistical tests showing awareness median score with participants’ characteristics (gender, age, province, educational level, and university status).

Variables	Awareness		
	Median	IQR	Value of p^*^
Gender			
Male	7.00	3	**0.004**
Female	8.00	2	
Age			
18–29	8.00	3	**< 0.001**
30–39	8.00	3	
40–49	8.00	3	
50–64	9.00	3	
65–74	7.5	3	
Province			
Beirut	8.00	2	0.243
Mount Lebanon	8.00	3	
Beqaa	8.00	3	
North Lebanon	7.50	3	
South Lebanon	8.00	2	
Baalbek – Hermel	8.00	3	
Akkar	7.00	3	
Nabatieh	8.00	2	
Educational level			
High School degree	7.00	2	**< 0.001**
Bachelor degree	8.00	3	
Master’s degree	8.00	2	
Pharm.D, Ph.D. degrees and postdoctoral fellowship	8.00	3	
University status			
Student	8.00	3	**0.003**
Staff	8.00	3	
Faculty	8.50	3	

*Values in bold indicate a significant value of *p* < 0.05.

### 3.3 Evaluation of practices

**Figure 2:**
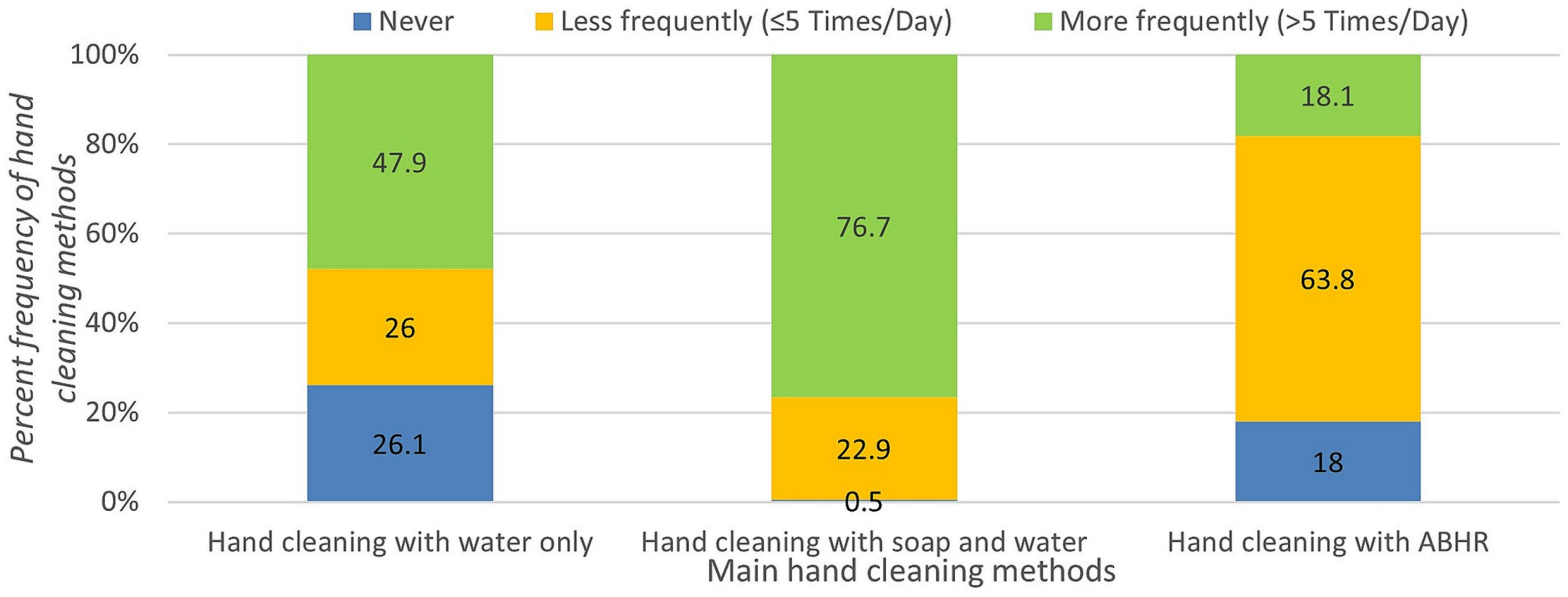
Percent frequency of hand cleaning methods applied by the study population.

According to Figure 2, the study population showed high commitment to the WHO hygiene recommendations (24) by reported handwashing with soap and water (99.5%) which was more frequently than ABHR (82%) and water only (73.9%). Table 4, which presents the statistical comparison of hand hygiene practices across gender, shows that females used water, ABHR or soap and water more frequently than males (*p* < 0.05), which highlights the necessity of raising the awareness of hand hygiene protocols specifically among males. Table 5 shows the frequency and percent frequency of hand hygiene practices across age. The results of the chi-square test indicate the preference of using soap and water among almost all age groups (*p* < 0.05). As indicated in Table 6, there is an association between the use of ABHR and province (*p* = 0.004 < 0.05). Education level does not show any association with water, water and soap or ABHR (*p* > 0.05, Table 7). This is contrary to the university status (academic, staff or student) which indicates significant differences for the use of water only (*p* < 0.05, Table 8).

**Table 4:** Frequency, percent frequency, and value of *p*s of hand hygiene practice methods based on gender.

Hand hygiene practices by gender	Never *n* (%)	Less frequently (≤ 5 times/day) *n* (%)	More frequently (> 5 times/day) *n* (%)	Value of p^*^
Water only				
Male	103 (21.9)	123 (26.2)	244 (51.9)	**0.024**
Female	234 (28.5)	213 (25.9)	374 (45.6)	
Soap and water				
Male	5 (1.1)	135 (28.7)	330 (70.2)	**< 0.001**
Female	1 (0.1)	160 (19.5)	660 (80.4)	
ABHR				
Male	102 (21.7)	282 (60.0)	86 (18.3)	**0.028**
Female	131 (16.0)	542 (66.0)	148 (18.0)	

^*^Values in bold indicate a significant value of *p* < 0.05.

**Table 5:** Frequency, percent frequency, and value of *p*s of hand hygiene practice methods based on age.

Hand hygiene practices by age	Never *n* (%)	Less frequently (≤ 5 times/day) *n* (%)	More frequently (> 5 times/day) *n* (%)	Value of p^*^
Water only				
18–29	271 (24.9)	289 (26.5)	530 (48.6)	0.542
30–39	47 (32.7)	32 (22.2)	65 (45.1)	
40–49	11 (30.6)	10 (27.7)	15 (41.7)	
50–64	7 (41.2)	4 (23.5)	6 (35.3)	
65–74	1 (25.0)	1 (25.0)	2 (50.0)	
Soap and water				
18–29	5 (0.5)	265 (24.3)	820 (75.2)	**< 0.001**
30–39	0 (0.0)	21 (14.6)	123 (85.4)	
40–49	0 (0.0)	6 (16.7)	30 (83.3)	
50–64	0 (0.0)	0 (0.0)	17 (100.0)	
65–74	1 (25.0)	3 (75.0)	0 (0.0)	
ABHR				
18–29	200 (18.4)	699 (64.1)	191 (17.5)	0.638
30–39	25 (17.4)	88 (61.1)	31 (21.5)	
40–49	6 (16.7)	22 (61.1)	8 (22.2)	
50–64	2 (11.8)	13 (76.4)	2 (11.8)	
65–74	0 (50.0)	2 (50.0)	2 (50.0)	

^*^Values in bold indicate a significant value of *p* < 0.05.

**Table 6:** Frequency, percent frequency, and value of *p*s of hand hygiene practice methods based on province.

Hand hygiene practices by province	Never *n* (%)	Less frequently (≤ 5 times/day) *n* (%)	More frequently (> 5 times/day) *n* (%)	Value of p^*^
Water only				
Beirut	68 (23.3)	86 (29.4)	138 (47.3)	0.053
Mount Lebanon	80 (33.1)	70 (28.9)	92 (38.0)	
Beqaa	37 (24.5)	38 (25.2)	76 (50.3)	
North Lebanon	43 (25.9)	36 (21.7)	87 (52.4)	
South Lebanon	60 (26.2)	52 (22.9)	116 (50.9)	
Baalbek – Hermel	12 (30.0)	8 (20.0)	20 (50.0)	
Akkar	21 (29.6)	15 (21.1)	35 (49.3)	
Nabatieh	16 (15.8)	31 (30.7)	54 (53.5)	
Soap and water				
Beirut	1 (0.3)	70 (24.0)	221 (75.7)	0.839
Mount Lebanon	0 (0.0)	56 (23.1)	186 (76.9)	
Beqaa	1 (0.7)	33 (21.8)	117 (77.5)	
North Lebanon	0 (0.0)	40 (24.1)	126 (75.9)	
South Lebanon	2 (0.9)	47 (20.6)	179 (78.5)	
Baalbek – Hermel	0 (0.0)	9 (22.5)	31 (77.5)	
Akkar	1 (1.4)	12 (16.9)	58 (81.7)	
Nabatieh	1 (1.0)	28 (27.7)	72 (71.3)	
ABHR				
Beirut	45 (15.4)	180 (61.6)	67 (23.0)	**0.004**
Mount Lebanon	26 (10.7)	162 (67.0)	54 (22.3)	
Beqaa	30 (19.9)	99 (65.6)	31 (20.5)	
North Lebanon	36 (21.7)	99 (59.6)	31 (18.7)	
South Lebanon	49 (21.5)	145 (63.6)	34 (14.9)	
Baalbek – Hermel	12 (30.0)	24 (60.0)	4 (10.0)	
Akkar	10 (14.1)	51 (71.8)	10 (14.1)	
Nabatieh	25 (24.7)	64 (63.4)	12 (11.9)	

^*^Values in bold indicate a significant value of *p* < 0.05.

**Table 7:** Frequency, percent frequency, and value of *p*s of hand hygiene practice methods based on education level.

Hand hygiene practices by education level	Never *n* (%)	Less frequently (≤ 5 times/day) *n* (%)	More frequently (> 5 times/day) *n* (%)	Value of p^*^
Water only				
High school degree	56 (20.4)	70 (25.6)	148 (54.0)	0.254
Bachelor degree	203 (27.2)	197 (26.3)	348 (46.5)	
Master’s degree	64 (28.3)	58 (7.7)	104 (14.0)	
Pharm.D, Ph.D. degrees and postdoctoral fellowship	14 (32.6)	11 (25.5)	18 (41.9)	
Soap and water				
High school degree	4 (1.5)	73 (26.6)	197 (71.9)	0.056
Bachelor degree	1 (0.1)	169 (22.6)	578 (77.3)	
Master’s degree	1 (0.4)	43 (19.0)	182 (80.6)	
Pharm.D, Ph.D. degrees and postdoctoral fellowship	0 (0.0)	10 (23.3)	33 (76.7)	
ABHR				
High School degree	54 (19.7)	168 (61.3)	52 (19.0)	0.132
Bachelor degree	132 (17.7)	494 (66.0)	122 (16.3)	
Master’s degree	44 (19.5)	133 (58.8)	49 (21.7)	
Pharm.D, Ph.D. degrees and postdoctoral fellowship	3 (7.0)	29 (67.4)	11 (25.6)	

**Table 8:** Frequency, percent frequency, and *p*-values of hand hygiene practice methods based on status.

Hand hygiene practices by status	Never *n* (%)	Less frequently (≤ 5 times/day) *n* (%)	More frequently (> 5 times/day) *n* (%)	*p*-value^*^
Water only				
Student	295 (25.6)	301 (26.2)	554 (48.2)	**0.008**
Staff	21 (48.8)	10 (23.3)	12 (27.9)	
Faculty	21 (21.4)	25 (25.5)	52 (53.1)	
Soap and water				
Student	6 (0.5)	268 (23.3)	876 (76.2)	0.559
Staff	0 (0.0)	6 (14.0)	37 (86.0)	
Faculty	0 (0.0)	21 (21.4)	77 (78.6)	
ABHR				
Student	216 (18.8)	735 (63.9)	199 (17.3)	0.125
Staff	5 (11.6)	28 (65.1)	10 (23.3)	
Faculty	12 (12.2)	61 (62.3)	25 (25.5)	

^*^Values in bold indicate a significant value of *p* < 0.05.

**Figure 3:**
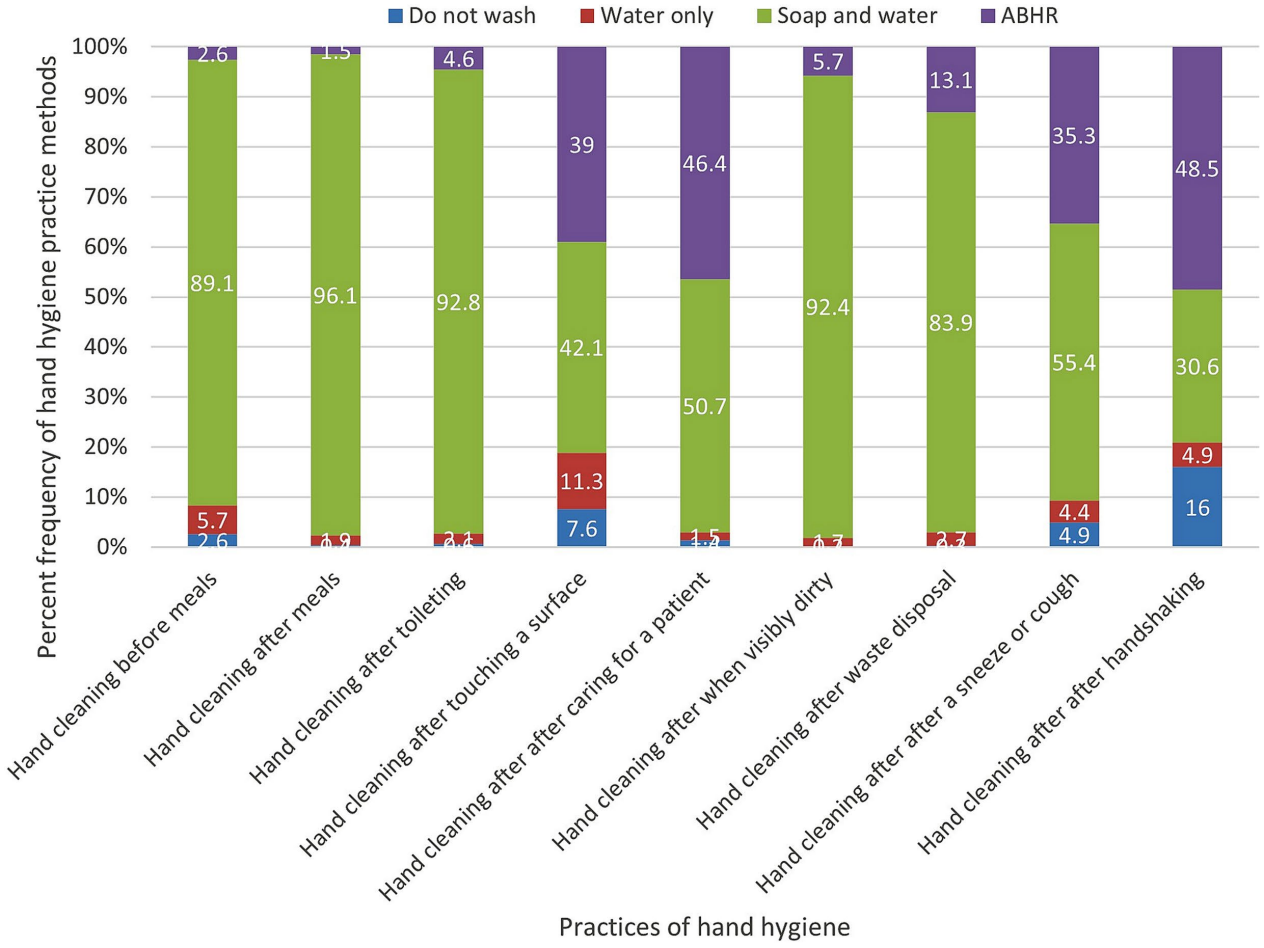
Percent frequency of hand hygiene practice methods using the nine actions.

Figure 3 represents the practices of hand hygiene reported by the study population before and after nine key actions. The participants showed high commitment to the CDC healthy habits (25) via reporting that soap and water was the predominant method of hand hygiene before meals (89.1%), after meals (96.1%), after toileting (92.9%), in the case of visibly dirty hands (92.5%), after waste disposal (83.9%), and after sneezing or coughing (55.4%). On the other hand, participants practiced comparably the methods of hand hygiene by ABHR with respect to soap and water after touching a solid surface (42.1% versus 39%), and after caring for a patient (50.7% versus 46.4%). The predominant use of ABHR for hand hygiene was reported only after shaking hands (48.5%).

## 4 Discussion

For effective prevention of the pandemic among the academic communities, this study was conducted to shed light on the awareness and practices of university communities regarding hand hygiene as one of the WHO-recommended preventive measures. To the best of our knowledge, no similar studies have been published among the Lebanese academic institutions. An anticipated outcome is to provide the Lebanese governmental authorities (MoPH and Ministry of Education and Higher Education, MEHE), the healthcare professionals, and public health researchers in Lebanon with new evidence on the hand hygiene, specifically awareness and practices. The study findings also provide a model to design educational campaigns to raise the awareness of hand hygiene and consequently limit the absenteeism among the university communities as a part of the United Nations (UN) sustainability goal of wellbeing (Sustainable Development Goal 3, SDG 3).

Worldwide, the fast spread of COVID-19 was a life-threating concern. To stop/limit the spread of the virus, governmental and non-governmental organizations have conducted several virtual campaigns to raise awareness of COVID-19 during the lockdown periods (26, 27). Consequently, public communities relied on multiple resources of information that may be reliable or non-reliable to get aware of the preventive measures of COVID-19. In this study, people preferred to get their information mostly from internet search engines and social media platforms; and to a less extent from the Lebanese MoPH, TV, healthcare professionals, and WHO/CDC webpages. However, their primary sources of information (internet webpages and social media platforms) may add a new concern regarding the unverified evidence and misleading information that could misguide people during the spread of the pandemics (28, 29). To enhance the passage of reliable information during the COVID-19 or any future pandemics, educational training and awareness campaigns should be developed virtually and on-site at academic institutions but also national levels (municipalities, factories and corporations, and entertainment centers) to become the most effective source of information during the pandemics.

In congruence with the Saudi Arabian study (23), participants showed a moderate level of hand hygiene awareness (score = 7.59 vs. 6.4 out of 12). The study finding indicates that most study participants (≥ 75% of the study population) had good awareness the role of hand hygiene to prevent the transmission of COVID-19, and other respiratory infections, but not HIV/AIDS. Poor-to-average awareness was detected in numerous aspects associated with the misconceptions of hand hygiene and proper handwashing techniques. For example, 42% were not aware that consistent handwashing does not affect body immunity (30), almost 40% were not aware that ABHR containing less than 60% of alcohol is not sufficient for hands disinfection (31), almost 69% were not aware that hands should be held underwater while lathering with soap (26), almost 52% were not aware that the temperature of the water does not make difference in terms of the cleansing effect of handwashing (26), and almost 71% were not aware that antiseptic/antibacterial soaps are no more effective at preventing illness than washing with plain soap and water (32). Concerningly, many participants were unaware that the high-risk practices (e.g., sanitizing bare hands with bleach or other household cleaners or by using ultra-violet lamps) can damage skin tissues (33). Anticipating such high-risk practices to prevent COVID-19 was observed in a population-based studies in the UAE and USA (34, 35). Similarly, literature studies focusing on university students showed awareness gap of the optimum temperature of water for effective handwashing and the necessary duration for hand disinfection (6, 14).

Comparing the awareness scores among the sociodemographic variables, females, and highly educated participants (university levels) recorded higher scores of awareness than males and high school graduates. This is in congruence with the studies investigating the public awareness toward the preventive measures of COVID-19 in Saudi Arabia (36), and the public awareness toward the use of household cleaners-disinfectants during the spread of pandemic (35). The finding may be explained by the fact that women are primarily responsible for taking care of their homes and maintaining high levels of hygiene at the personal and household levels (37). In the other notable variable (impact of education), education level plays an important role in raising the level of awareness concerning preventive measures and hygiene products of COVID-19 as explained in the previous published studies (37–39). This hypothesis was also confirmed in the university status when the faculty members (highly educated participants) recorded a higher score of awareness than the less educated participants (university students and administrative staff). Additionally, the age variable plays a significant role in raising the awareness toward hand hygiene among the study population where participants below 65 years old showed higher scores than other older adult participants (aged ≥65 years old). This finding may be explained that people with younger age groups are more involved in COVID-19 campaigns about either prevention or treatment of the infected people than older adult people (40).

Focusing on the hand hygiene practices, the study population reported handwashing with soap and water more frequently than other protocols (ABHR or water) due to their strong beliefs that cleansing hands with soap and water is the most effective method (23). Similar to other studies (6, 8, 14, 23), the predominant practices which were reported by the study population before and after nine key actions were handwashing with soap and water before meals, after meals, after toileting, when hands visibly dirty, after waste disposal, and after sneezing or coughing. On the other hand, ABHR or soap and water were comparably utilized after touching a solid surface, after caring for a patient when compared to the Saudi Arabian community that showed a comparable methods (ABHR, soap and water) after caring for a patient, and after handshaking (23). Overall, the study population showed high compliance of hand hygiene recommendations of CDC to prevent the spread of the pandemic (41).

In this study protocol, several limitations must be noted. The study finding may be biased by the self-responding feature of the utilized tool and the uneven distribution of the study population (dominant number of students and females). The online data collection during the spread of the pandemic could also limit the randomness of the study sampling. To overcome this limitation, potential participants were recruited through social media platforms in addition to distributing the study tool via the research departments of the Lebanese academic institutions. Based on the study findings and limitations, community-based interventions and research-based assessments are highly recommended to ensure the full compliance to hand hygiene guidance among public, academic, and occupational communities in Lebanon.

## 5 Conclusion

In Lebanon, university communities (faculty, students, and staff) showed an average levels awareness highlighting the necessity of training programs to promote the appropriate technical skills of hand hygiene. Associated variables such as gender, age, educational level, and university status contributed positively to awareness level among the university communities. Awareness campaigns and health education programs are recommended to raise the awareness and skills of hand hygiene among the academic communities of Lebanon.

## Data availability statement

The original contributions presented in the study are included in the article/supplementary material, further inquiries can be directed to the corresponding authors.

## Ethics statement

The studies involving humans were approved by the Institutional Review Board (IRB) at the Modern University for Business and Science (MUBS) approved the ethical application (MU20210924-25). The studies were conducted in accordance with the local legislation and institutional requirements. The participants provided their written informed consent to participate in this study.

## Author contributions

NA: Conceptualization, Data curation, Formal analysis, Methodology, Project administration, Supervision, Writing – original draft, Writing – review & editing. JS: Data curation, Methodology, Project administration, Writing – review & editing. RK: Data curation, Methodology, Project administration, Writing – review & editing. WG: Conceptualization, Data curation, Formal analysis, Methodology, Project administration, Supervision, Writing – original draft, Writing – review & editing.
